# What Motivates Chinese Young Adults to Use mHealth?

**DOI:** 10.3390/healthcare7040156

**Published:** 2019-12-02

**Authors:** Wai-Ming To, Peter K. C. Lee, Jinxuan Lu, Junhao Wang, Yihan Yang, Qingxin Yu

**Affiliations:** 1 School of Business, Macao Polytechnic Institute, Macao SAR 999078, China; wmto@ipm.edu.mo; 2 Department of Logistics and Maritime Studies, The Hong Kong Polytechnic University, Hong Kong SAR 999077, China; rainydeer1231@gmail.com (J.L.); jackiwjh@icloud.com (J.W.); yangyihan1997@gmail.com (Y.Y.); joyceqx815@gmail.com (Q.Y.)

**Keywords:** mHealth, technology acceptance, communication effectiveness, Chinese, young adults

## Abstract

mHealth is one of China’s national strategies that brings affordable, accessible, and convenient health care to its entire population, may they be in cities or rural areas. Although Chinese young adults are among the first to adopt mHealth, the factors influencing Chinese young adults to use mHealth are yet to be studied both empirically and in depth. This study explores the mechanism that determines Chinese young adults’ intention to use mHealth, based on an extended Technology Acceptance Model (TAM). The extended TAM was tested using responses from 486 Chinese young adults. The results showed that perceived usefulness strongly and significantly influenced people’s intention to use mHealth. Additionally, communication effectiveness, health consciousness, and perceived ease of use were found as significant factors influencing people’s intention to use mHealth through perceived usefulness. Distrust was not found to significantly influence people’s intention to use mHealth.

## 1. Introduction

According to the World Health Organization [1], mHealth refers to the deployment and application of mobile technologies for the delivery of public health. mHealth encompasses a wide range of mobile technologies that address people’s health care needs such as access to health information, medical advice, health-care services, and medical follow-up [2,3]. Specifically, mHealth views patients as consumers and empowers them to manage their own health [4]. In the US, around one third of cell phone users have accessed health information, including behavioral health issues, frequently using mobile devices [5,6]. In China, mHealth has been used to improve communications between patients and health care providers, promote public health awareness and health education, and enhance the efficiency of medical services and public health campaigns [7,8]. mHealth is becoming more and more important because China’s number of mobile Internet users has increased from 346 million in 2011 to 817 million in 2018 [9,10], and the number of active mHealth users was 466 million in the first quarter of 2019 [11]. About 82.3% of mHealth users regularly access online medical services, 71.8% for health management information services, 55.6% for medical care information, and 32.4% for drug and pharmaceutical services [11]. The largest mHealth service provider in China, Ping An Good Doctor [12], reported that it has over 265 million registered users and offers a wide range of services to its users from mobile medical consultations, mobile drug purchases to mobile follow-up services. The number of mobile users for “Yeemiao”, an App for vaccination that was launched in 2014, increased rapidly from 5 million in March of 2017 to over 20 million in April of 2019. Yeemiao links users to over 28,000 hospitals and clinics all over China and provides information on maternal and child health. Thus, mHealth is defined as the provision of health information and services via mobile technologies, as suggested by Hsu et al. [13].

Gagnon et al. [14] investigated the adoption of mHealth among health professionals. Based on a systematic review approach, they reported that perceived ease of use, perceived usefulness, cost, security, privacy, and interaction with others influenced health professionals’ adoption of mHealth. Hoque and Sorwar [15] studied motivational factors for the use of mHealth among the elderly in developing countries. Using responses from 274 people aged 60 or above in Bangladesh, they reported that effort expectancy (i.e., perceived ease of use), social influence, and performance expectancy (i.e., perceived usefulness) positively influenced people’s intention to adopt mHealth services. People’s behavioral intention, on the other hand, was negatively affected by technology anxiety and resistance to change [15]. Jen and Hung [16] investigated the adoption of mobile health care services for Taiwanese elderly people. By integrating the Technology Acceptance Model (TAM) and Theory of Planned Behavior, it was found that perceived ease of use and perceived usefulness indirectly influenced people’s intention to use mobile health services for their elderly family members through attitude towards technology [16]. Lee et al. [17] explored context- and content-related factors affecting people’s intention to use mHealth. Using responses from 313 South Koreans aged over 40, they reported that perceived usefulness and convenience significantly influenced people’s intention to use mHealth. Zhang et al. [18] studied various factors affecting the adoption of mHealth and explored whether gender had an effect on mHealth adoption in north China. They reported that subjective norm had the greatest effect on mHealth adoption intention, followed by facilitating conditions and attitude towards mHealth. Zhang et al. [18] also showed that women had slightly more positive attitudes and higher intentions to adopt mHealth, and they indicated that some other factors might also influence mHealth adoption intention. Lu et al. [19] examined the user demographics of mHealth in 2016. They reported that the majority of mHealth users in China are young adults who are well educated and live in urban areas. mHealth users also have a greater demand for specialist medical services. As Chinese young adults are among the first to use mHealth and will help their elderly relatives to embrace mHealth, this study aims to answer the following research question: What drives users to adopt mHealth in China these days, particularly with Chinese young adults who are constantly exposed to ever-changing mobile technologies? 

Based on the widely used TAM, the present study investigates whether and how perceived ease of use and perceived usefulness influence people’s intention to use mHealth. Additionally, the study explores the effects of health consciousness, communication effectiveness, and distrust on people’s intention to use mHealth, both directly and indirectly through perceived usefulness. 

## 2. Literature Review and Hypothesis Development 

TAM is one of the most widely used theoretical models that predicts the acceptance of information systems and communication technologies by individual users [20,21]. Over the years, TAM and its extended versions have been used to predict people’s adoption of email [22,23,24], word processing and spreadsheet systems [25,26], e-commerce [27,28], social media such as Facebook, WhatsApp, Twitter, and WeChat [29,30,31], mobile fashion apps [32], personal digital assistants (PDAs) in health care [33], and mHealth [15,34]. According to Davis [22], individuals’ intention to use a specific information technology depends on perceived ease of use (i.e., effort expectancy) and perceived usefulness (i.e., performance expectancy). In Davis’ terminology [22,23], perceived ease of use measures the degree to which individuals believe that using technology is free of effort while perceived usefulness measures the degree to which individuals believe that using technology would enhance work performance. Individuals’ actual use of technology is determined by individuals’ intention [23]. Additionally, perceived ease of use influences individuals’ perception of the usefulness of technology [22,23]. Thus, we propose the following three basic hypotheses:

**Hypothesis** **1.** *Perceived ease of use positively influences people’s intention to use mHealth.*

**Hypothesis** **2.** *Perceived usefulness positively influences people’s intention to use mHealth.*

**Hypothesis** **3.** *Perceived ease of use positively influences perceived usefulness of mHealth.*

The original TAM has limitations (even though the original TAM’s strength was its simplicity). For this reason, Bogozzi [35] and Benbasat and Barki [36] informed fellow researchers that they should start looking for contextual issues and psychological drivers or antecedents that influence people’s beliefs. Perceived ease of use and perceived usefulness are individual dispositions toward technology (i.e., mHealth in this case). They are influenced by individual and contextual factors. Among different individual factors, health consciousness—referring to the degree to which individuals take care of their own health—was found to positively influence people’s online health information seeking behavior [37]. Cho et al. [38] explored individuals’ cognitive factors of using health apps. Using responses from 765 Korean adults, Cho et al. [38] found that health consciousness was the most significant predictor to adopting health apps, while eHealth literacy and health information orientation did not directly influence the adoption of health apps. Ahadzadeh et al. [39], and Yun and Park [40] examined the impact of health consciousness on health-related Internet use. They reported that health consciousness positively and significantly affected people’s perceived usefulness. Hence, we posit that:

**Hypothesis** **4.** *Health consciousness positively influences perceived usefulness of mHealth.*

**Hypothesis** **5.** *Health consciousness positively influences people’s intention to use mHealth.*

Communication effectiveness is a context-related belief. It refers to the degree to which users believe that technology can transmit information accurately and effectively between information providers and them [41,42]. Lu et al. [43] studied the factors influencing the adoption of short message service (SMS) in China. They suggested that communication effectiveness could affect perceived usefulness and people’s adoption of SMS. However, their survey results showed that communication effectiveness significantly influenced the adoption of SMS but did not have a significant effect on perceived usefulness. Nevertheless, researchers [7,8] indicated that communication effectiveness facilitates interactions between people and health care providers, thus influencing perceived usefulness and people’s intention to use mHealth. Thus, we posit that:

**Hypothesis** **6.** *Communication effectiveness positively influences perceived usefulness of mHealth.*

**Hypothesis** **7.** *Communication effectiveness positively influences people’s intention to use mHealth.*

Trustworthiness is another major contextual issue as information and communications technologies become ubiquitous and pervasive [44,45]. This is especially true for highly customized or very personal services such as mHealth [44]. Distrust is not only the absence of trust; it refers to people’s negative expectations that the other party such as a website will behave in a way that harms the interests of users and causes skepticism [46]. Ou and Sia [46] reported that distrust significantly and negatively influenced people’s online purchase intention. Chang and Fang [47] studied the effects of distrust and trust on the Internet behaviors. Using responses from 1153 Taiwanese, Chang and Fang [47] reported that distrust had a more significant effect on reducing the amount of high-risk Internet behavior than trust did on enhancing it. Hence, we posit that:

**Hypothesis** **8.** *Distrust negatively influences people’s intention to use mHealth.*

**Figure 1:**
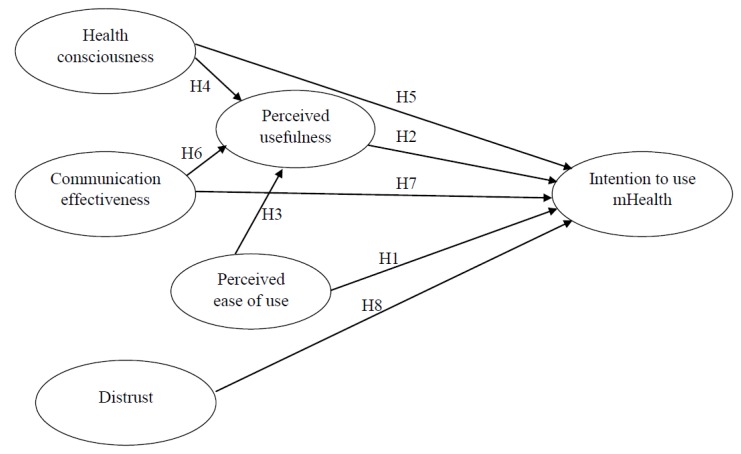
Theoretical model of the study.

Figure 1 shows the relationships between perceived ease of use, perceived usefulness, and people’s intention to use mHealth. As an extended TAM model that incorporates antecedents of perceived usefulness and people’s intention in the mHealth context, Figure 1 presents how health consciousness and communication effectiveness may influence perceived usefulness directly and people’s intention directly and indirectly via perceived usefulness. Figure 1 also indicates how distrust may negatively influence people’s intention to use mHealth.

## 3. Materials and Methods 

### 3.1. Study Design and Sample

A survey based on a cross-sectional questionnaire was conducted to obtain people’s views on mHealth. We adopted two types of data collection methods, namely face-to-face questionnaire administration and online questionnaire survey via two of the largest Chinese social media platforms i.e., Weibo and WeChat. We used a convenience sampling approach and contacted 1412 respondents in China. Among all respondents, 486 respondents who were categorized as young adults aged from 20 to 39 years [48] completed and returned valid responses to the survey, resulting in a usable response rate of 34.4%.

### 3.2. Instrument

The questionnaire had three parts. The first part had six questions to collect demographic characteristics of respondents such as gender, age, employment status, annual income or family support, medical coverage, and hours spent on mobile phone per day. The second part contained 17 items measuring six constructs of the theoretical model. As a reminder to respondents, we indicated mHealth as the provision of health information and services via mobile technologies such as mobile health apps offered by Ping An Good Doctor, Yeemiao, etc. Health consciousness measures the degree to which individuals are concerned about their own health and wellbeing. We adapted three items from Ahadzadeh et al. [39]. Communication effectiveness measures the degree to which individuals believe that technology can pass on information accurately and effectively between service providers and themselves. We adapted three items from Park et al. [41]. Distrust measures the degree to which individuals view technology negatively, signaling fear, worry, and skepticism. We adapted three items from Akter et al. [44] and Ou and Sia [46]. Perceived ease of use measures the effort individuals foresee in using technology. We adapted three items from Jen and Hung [16]. Perceived usefulness measures the degree to which individuals believe that using technology would enhance work performance. We adapted three items from Jen and Hung [16]. Intention to use measures the degree to which individuals believe that they plan to use technology. We adapted two items from Hur et al. [32]. All items were rated using a five-point Likert scale, with 1 representing “strongly disagree” and 5 representing “strongly agree”.

Table 1 shows items belonging to the six core constructs. The Cronbach’s alpha values for health consciousness, communication effectiveness, distrust, perceived ease of use, perceived usefulness, and intention to use mHealth were 0.89, 0.79, 0.81, 0.78, 0.87, and 0.87, respectively. They were all higher than the threshold level of 0.70, supporting that the constructs are reliable [49]. The third part contained an open-ended question to explore the motivational factors that might influence respondents’ intention to use mHealth. 

The questionnaire was developed in English. It was translated to Chinese by a bilingual researcher and was then back-translated by another independent bilingual professional. The two English versions of the questionnaire were cross-checked to ensure the consistency of survey items [50]. The questionnaire was pilot-tested on a group of 40 respondents. These respondents indicated that the questionnaire was easy to follow and instructions and items were clear, supporting content validity. 

## 4. Results

Among 486 respondents, 56.6% (275/486) were female. Most respondents (54.9%) were of age 20–24 years, followed by the age group 25–29 years (23.3%). Most respondents were employed (52.5%), followed by students (43.6%). A total of 34.1% of respondents had an annual income or family support less than RMB 80,000, 29.6% between RMB 80,000 and RMB 150,000, and 32.1% between RMB 150,000 and RMB 800,000. The majority of respondents (84%) had medical insurance. In terms of hours spent on mobile phone per day, most respondents (34.0%) selected 4–6 hours, followed by 6–8 hours (27.2%). This finding was consistent with what was reported in China Daily—the average Chinese mobile user spent around 4.7 hours per day in using his/her mobile apps in the second quarter of 2019 [51]. Table 2 presents the demographic profile of respondents. 

A series of *t*-tests and analyses of variance (ANOVAs) were performed to explore the effect of gender, age, income, and time spent in using mobile phone on responses on questionnaire items. Results indicated that there were no significant effects of gender, age, and time spent in using mobile phone on most items. However, the annual income did have some significant effects on item responses. Table 3 shows the means and standard deviations of items for different income groups. Results from ANOVA revealed that people with an annual income of RMB 0.8 million or above were more health conscious and had higher ratings on items of communication effectiveness, perceived ease of use, perceived usefulness, and intention to use mHealth.

Table 4 shows the means, standard deviations, and correlations between the constructs used in the study. It was found that health consciousness was moderately associated with communication effectiveness (r = 0.40; *p* < 0.01), and strongly associated with perceived ease of use (r = 0.56; *p* < 0.01), perceived usefulness (r = 0.59; *p* < 0.01), and people’s intention to use mHealth (r = 0.56; *p* < 0.01). Communication effectiveness was moderately associated with perceived ease of use (r = 0.45; *p* < 0.01), and strongly associated with perceived usefulness (r = 0.52; *p* < 0.01) and people’s intention to use mHealth (r = 0.53; *p* < 0.01). Perceived ease of use was strongly associated with perceived usefulness (r = 0.71; *p* < 0.01) and people’s intention to use mHealth (r = 0.57; *p* < 0.01). Perceived usefulness was strongly associated with people’s intention to use mHealth (r = 0.75; *p* < 0.01). On the other hand, distrust was found to be weakly associated with health consciousness (r = 0.19; *p* < 0.01) and perceived ease of use (r = 0.16; *p* < 0.01).

### 4.1. Confirmatory Factor Analysis and Structural Equation Modeling

Confirmatory factor analysis was performed with IBM SPSS Amos 26 [52]. A six-factor measurement model comprising health consciousness, communication effectiveness, distrust, perceived ease of use, perceived usefulness, and intention to use mHealth produced a chi-square value (*χ*^2^) of 274.3 with 104 degrees of freedom (*df*). The normed chi-square statistic (*χ*^2^/*df*) was 2.637, indicating a good fit between the model and data [53]. Additionally, the model produced three other indices: non-normed fit index (NNFI) = 0.95, comparative fit index (CFI) = 0.97, and root mean square error of approximation (RMSEA) = 0.06. All of them met the good fit criteria for NNFI ≥ 0.95, CFI ≥ 0.95, and RMSEA ≤ 0.05 as suggested by Hair et al. [53], and Hu and Benter [54]. The factor loadings of items ranged from 0.65 to 0.96, except that the first item of perceived ease of use, i.e., PEOU1 only had a factor loading of 0.49. After dropping this item, the measurement model with six constructs and 16 items produced the following fit indices: *χ*^2^/*df* = 2.036 (*χ*^2^ = 181.2; *df* = 89), NNFI = 0.96, CFI = 0.98, RMSEA = 0.05, all meeting the criteria as stated above. Factor loadings ranged from 0.65 to 0.96, as shown in Table 1. Table 1 also presents the composite reliabilities and average variance extracted (AVE) values for the six constructs. The values of composite reliability ranged from 0.80 to 0.90, all above the threshold of 0.7, as suggested by Nunnally and Bernstein [49]. The values of AVE ranged from 0.57 to 0.74, all above the threshold of 0.5 as suggested by Nunnally and Bernstein [49]. All these supported the reliability of the six constructs. The values of the square root of AVE are shown in the diagonal elements of Table 4. It was found that the value of the square root of AVE of a construct was greater than the correlations between that construct and all other constructs, supporting discriminant validity [55]. 

To check the extent of common method variance and the possibility of an alternative model, we tested a second measurement model with one factor and 16 items. This one-factor measurement model produced the following fit indices: *χ*^2^/*df* = 18.6 (*χ*^2^ = 1934.4; *df* = 104), NNFI = 0.61, CFI = 0.63, RMSEA = 0.19, indicating that this one-factor model did not fit the collected data well. Thus, the six-factor model was considered appropriate. 

**Figure 2:**
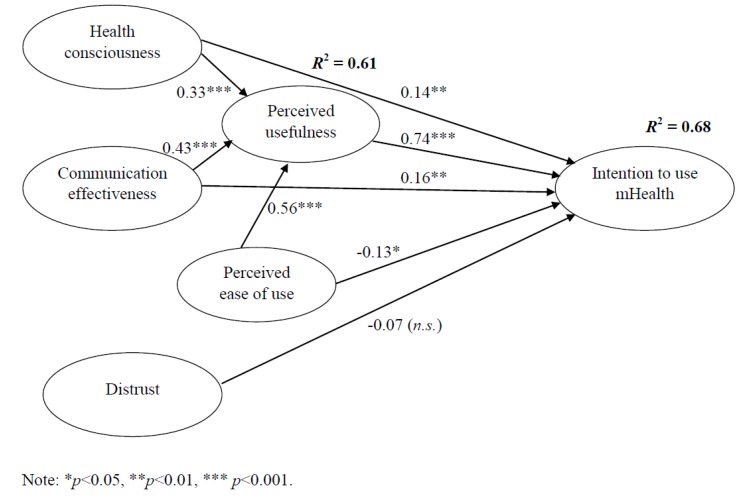
The final structural model.

Structural equation modeling was performed with IBM SPSS Amos 26 using maximum likelihood estimation. The structural model produced the following fit indices: *χ*^2^/*df* = 4.928 (*χ*^2^ = 473.1; *df* = 196), NNFI = 0.91, and CFI = 0.92. These indices indicated that the structural model was considered acceptable [53]. The value of RMSEA was 0.09, which could be considered as marginally acceptable [53]. Figure 2 shows the final structural model. Table 5 shows the direct, indirect, and total effects of health consciousness, communication effectiveness, perceived ease of use, perceived usefulness, and distrust on people’s intention to use mHealth. This table also shows the direct effects of health consciousness, communication effectiveness, and perceived ease of use on perceived usefulness. Overall, it was found that perceived usefulness strongly and significantly influenced people’s intention to use mHealth (β = 0.74; *p* < 0.001), supporting Hypothesis 2. Additionally, perceived usefulness was strongly and significantly affected by perceived ease of use (β = 0.56; *p* < 0.001), health consciousness (β = 0.33; *p* < 0.001), and communication effectiveness (β = 0.43; *p* < 0.001), supporting Hypothesis 3, Hypothesis 4, and Hypothesis 6, respectively. Both health consciousness (β = 0.14; *p* < 0.05) and communication effectiveness (β = 0.16; *p* < 0.05) significantly affected people’s intention to use mHealth moderately, supporting Hypothesis 5 and Hypothesis 7, respectively. On the other hand, the direct influence of perceived ease of use on people’s intention to use mHealth was significant but negative (β = −0.13; *p* < 0.01), rejecting Hypothesis 1. However, the total effect of perceived ease of use on people’s intention to use mHealth was positive because perceived ease of use had a significant, indirect, and greater effect on people’s intention to use mHealth through perceived usefulness, as shown in Table 5. Distrust did not show significant effect on people’s intention to use mHealth (β = −0.07; *n.s.*), rejecting Hypothesis 8. In terms of the total effects, the most significant predictor of people’s intention to use mHealth was communication effectiveness, followed by health consciousness and perceived ease of use. The results of hypothesis testing are summarized in Table 6.

### 4.2. Key Themes from Open-Ended Questions

Based on the qualitative data collected from the open-ended question concerning the motivational factors that influence respondents’ intention to use mHealth, we categorized the responses under the following areas: provide more ways to improve real-time and accurate communication between patients and doctors (frequency: 68); assist patients to describe their symptoms more accurately (frequency: 56); reduce advertisements and show more user experiences (frequency: 53); provide opportunities for better responsive behavior for doctors (frequency: 47); provide better healthcare education (frequency: 42); provide more channels to see users’ comments and complaints (frequency: 36); complement diagnosis with comprehensive healthcare information (frequency: 20); and provide information about support group, i.e., patients with similar disease or treatment (frequency: 15). Thus, the main themes were concerns about communication effectiveness between users and doctors, followed by information that can improve health consciousness.

## 5. Discussion

mHealth is a very important strategy to provide nationwide accessible health services to people. It is particularly crucial to a huge developing country such as China. This paper explored key factors influencing Chinese young adults’ intention to use mHealth using the extended TAM. The results showed that communication effectiveness and health consciousness moderately and significantly influenced people’s intention to use mHealth, both directly and indirectly through perceived usefulness with total effects of 0.48 (= 0.16 + 0.43 × 0.74) and 0.38 (= 0.14 + 0.33 × 0.74), respectively. Communication effectiveness was found to be the most important antecedent, similar to the study of Kim et al. [56], which identified engagement, content quality, and reliability to be key factors influencing people’s intention to continue to use mHealth in Korea. Surprisingly, the mean score of health consciousness among Chinese young adults was only 1.66 on a five-point Likert scale, with 1 meaning “strongly disagree” and 5 meaning “strongly agree”. Health consciousness was found to be the second most important factor influencing people’s intention to use mHealth. As the Chinese government continues to invest heavily in the healthcare sector and promote a healthy lifestyle at an unprecedented rate [57], it is expected that health consciousness among Chinese young adults will improve continually in the near future. Perceived ease of use significantly influenced people’s intention to use mHealth through perceived usefulness. Thus, maintaining simplicity and ease of use can greatly enhance perceived usefulness of mHealth. Overall, perceived usefulness was found to play the most important role in shaping people’s intention to use mHealth. This finding was consistent with what Mellikeche et al. [58] reported in their study on other health-related information systems in hospitals. It should be noted that the total effect of perceived ease of use on people’s intention was 0.29 and the direct and indirect effects of perceived ease of use on people’s intention were −0.13 and 0.42 (= 0.56 × 0.74), respectively. Yet, why the direct effect of perceived ease of use on people’s intention was negative warrants further investigation. On the other hand, distrust was found not to have a significant effect on people’s intention to use mHealth. The Chinese government has passed new laws and regulations in recent years to ensure that health care providers and the Internet search engines shall provide accurate information on medical-related services and products and the collusion between health care providers and pharmaceutical companies is prohibited, particularly after the “Wei Zexi” incident [59]. Hence, the problem of distrust seems to have diminished in the past few years. 

### 5.1. Limitations and Future Research

This study has several limitations. First, this study adopted a cross-sectional dataset (i.e., collecting responses from the target population at a specific point in time) to test the posited hypotheses. Thus, causality between variables in hypotheses is a potential issue. Future work may collect and use longitudinal data to address this issue. Second, this study used self-reported measures and responses might be affected by common method variance. Harman’s one factor test was conducted to check for the extent of common method variance [60]. The unrotated principal component factor analysis showed that no single factor emerged, and the first factor accounted for 42.6% (less than 50%) of the total variance. Additionally, the fit indices for the one-factor measurement model were much poorer than that for the six-factor measurement model, suggesting that common method variance was unlikely to be a serious issue. Third, the research context of this study is China, which is a developing country. Specifically, many Chinese mobile users spend four or more hours a day using their mobile phones [51] and could be considered as heavy mobile phone users. Yet, their ratings of perceived ease of use and perceived usefulness were 2.38 and 2.32, as seen in Table 4, which were much lower than 3.0—the midpoint of the five-point Likert scale—even though perceived ease of use and perceived usefulness significantly influenced people’s intention to use mHealth among Chinese young adults. Internet users of countries with differing levels of economic development may behave differently in the ways they use apps. Future work may examine Internet users of different countries to examine if there is a relationship between economic development and people’s intention to use mHealth. Finally, the theoretical view of this study is TAM. Future work may use other relevant theories (e.g., innovation diffusion) to identify and examine other predictors of the intention to use mHealth.

### 5.2. Practical Implications

Our findings indicate that the most critical factor influencing people to use mHealth is the usefulness of the app. Our findings also indicate that, to improve mHealth app’s usefulness, mHealth providers should pay extra attention to three important aspects: (1) Communication between patients and doctors must be effective; (2) health-related information must be promoted; and (3) the app should be easy to use. Thus, mHealth’s providers must be willing to invest resources in improving app design. For instance, mHealth providers can improve their app design using the responses from our questionnaire. Responses such as “improving real-time and accurate communication”, “reducing advertisements”, “having better healthcare education”, and “having channels to see users’ comments and complaints”, provide useful and relevant insights, which can help mHealth to better design their apps. Additionally, the apps should have features listing choices of symptoms of major diseases with scales for patients to indicate the degree of illness and symptoms (e.g., pain, dizziness). Second, another factor that encourages people to use mHealth is people’s health consciousness. Thus, mHealth providers can use different means to make people understand the importance of maintaining good health. Specifically, they can do that through their apps, their company webpages on the Internet, and different social media platforms. Finally, the app design should be user friendly and structured in a way that enables future modifications and extensions.

## 6. Conclusions

This paper explored key factors influencing Chinese young adults’ intention to use mHealth. Using the extended TAM and responses from 486 Chinese young adults, we found that communication effectiveness, health consciousness, and perceived ease of ease significantly influenced people’s intention to use mHealth directly and indirectly through perceived usefulness. Specifically, communication effectiveness was found to be the most significant predictor of people’s intention, followed by health consciousness. 

## Figures and Tables

**Table 1:** Constructs’ items with factor loadings, composite reliability (CR), and average variance extracted (AVE).

Construct and Items	Factor	CR	AVE
Health consciousness (HC)		0.90	0.76
HC1—I think it is important to know well about how to stay healthy	0.91		
HC2—I think I should take health into account a lot in my life.	0.96		
HC3—I ask myself at the time whether the things I eat are healthy for me.	0.73		
Communication effectiveness (EC)		0.80	0.57
CE1—I shall be able to describe my symptoms of illness thoroughly and clearly to doctors through mHealth.	0.65		
CE2—Doctors on mHealth are able to diagnose my disease accurately and give me suitable advice according to the information I provide.	0.86		
CE3—My communication with doctors on mHealth shall be as effective as going to hospitals and seeing doctors physically.	0.74		
Distrust (DIS)		0.82	0.60
DIS1—mHealth may not have enough professionalism and competence in providing medical service.	0.69		
DIS2—I am concerned about the reliability of mHealth.	0.85		
DIS3—I am worried about relying on mHealth.	0.77		
Perceived ease of use (PEOU)		0.88	0.78
*PEOU1*—mHealth is flexible to interact with.^1^	*0.49*		
PEOU2—Learning to operate mHealth is easy for me.	0.85		
PEOU3—It is easy to become skillful at using mHealth.	0.94		
Perceived usefulness (PU)		0.87	0.69
PU1—Using mHealth is beneficial to my health.	0.84		
PU2—The functions of mHealth make my life more convenient.	0.84		
PU3—In general, I think that the advantages of mHealth outweigh the disadvantages.	0.81		
Intention to use mHealth (INT)		0.87	0.78
INT1—I am interested in trying to use mHealth.	0.86		
INT2—I plan to use mHealth in the near future.	0.90		

Notes: A factor loading is the correlation between the item and the underlying construct (the cut-off threshold is 0.5). Composite reliability describes the extent to which the items represent a common underlying construct (the acceptable value is 0.70 and above). Average variance extracted shows the amount of variance captured by the measurement items for the underlying construct (the acceptable value is 0.50 or above); ^1^ This item (*PEOU1*) was dropped because its factor loading was only 0.49, less than 0.50.

**Table 2:** Demographic profile of respondents (*N* = 486).

Variable	Class	Frequency	Percent
Gender	Male	211	53.4
	Female	275	56.6
Age	20–24	267	54.9
	25–29	113	23.3
	30–34	69	14.2
	35–39	37	7.6
Status	Student	212	43.6
	Employed	255	52.5
	Self-employed	12	2.5
	Others	7	1.4
Annual income or family support (in thousands RMB)	<30	40	8.2
	30–80	126	25.9
	80–150	144	29.7
	150–800	156	32.1
	>800	20	4.1
Medical insurance	Covered	408	84
	Uncovered	35	7.2
	Unclear	43	8.8
Time spent on mobile phone per day (hours)	<2	10	2.1
	2–4	120	24.7
	4–6	165	34
	6–8	132	27.2
	>8	59	12.1

**Table 3:** Means and standard deviations (SD) of items for different income groups.

Item	Annual Income or Family Support (in Thousand RMB)				
	<30	30–80	80–150	150–800	800+
	Mean (SD)	Mean (SD)	Mean (SD)	Mean (SD)	Mean (SD)
HC1	1.55 (0.597)	1.48 (0.678)	1.54 (0.938)	1.49 (0.807)	2.25 (1.618)
HC2	1.43 (0.549)	1.40 (0.622)	1.60 (0.956)	1.52 (0.807)	2.25 (1.517)
HC3	1.85 (0.802)	1.68 (0.876)	1.99 (0.993)	1.94 (1.011)	2.40 (1.353)
CE1	2.98 (1.097)	2.78 (0.884)	2.79 (0.960)	3.06 (0.883)	3.60 (0.883)
CE2	3.03 (0.891)	2.71 (0.838)	2.95 (0.847)	3.04 (0.773)	3.60 (0.940)
CE3	3.18 (0.984)	3.06 (0.851)	3.10 (0.926)	3.43 (0.951)	3.85 (0.754)
PEOU2	2.45 (0.876)	2.26 (0.761)	2.29 (0.860)	2.10 (0.841)	2.90 (1.119)
PEOU3	2.23 (0.800)	2.17 (0.713)	2.24 (0.836)	2.01 (0.884)	2.85 (1.309)
PU1	2.48 (0.751)	2.30 (0.673)	2.33 (0.802)	2.27 (0.798)	2.90 (1.071)
PU2	2.28 (0.640)	2.19 (0.654)	2.29 (0.801)	2.21 (0.803)	2.70 (0.865)
PU3	2.48 (0.816)	2.33 (0.747)	2.38 (0.836)	2.29 (0.887)	2.80 (1.281)
INT1	2.40 (0.810)	2.29 (0.716)	2.42 (0.920)	2.58 (0.894)	3.10 (1.252)
INT2	2.42 (0.781)	2.24 (0.686)	2.33 (0.853)	2.44 (0.844)	2.90 (1.210)

Notes: HC stands for health consciousness, CE stands for communication effectiveness, PEOU stands for perceived ease of use, PU stands for perceived usefulness, and INT stands for intention to use mHealth. It should also be noted that there was no significant difference between different income groups for items of distrust (DIS).

**Table 4:** Means, standard deviations (SD), and correlations between the constructs.

Construct	Mean (SD)	HC	CE	DIS	PEOU	PU	INT
Health consciousness (HC)	1.66 (0.810)	***0.87***					
Communication effectiveness (CE)	3.04 (0.767)	0.40 **	***0.75***				
Distrust (DIS)	2.18 (0.690)	0.19 **	−0.02	***0.77***			
Perceived ease of use (PEOU)	2.38 (0.703)	0.56 **	0.45 **	0.16 **	***0.89***		
Perceived usefulness (PU)	2.32 (0.711)	0.59 **	0.52 **	0.09	0.71**	***0.83***	
Intention to use mHealth (INT)	2.42 (0.807)	0.56 **	0.53 **	0.03	0.57 **	0.75 **	***0.88***

Notes: The construct’s mean is the grand mean response of the measurement items. The construct’s standard deviation is calculated using the mean responses of the measurement items; ** *p* < 0.01. Italic bold values on the diagonal are square roots of AVE.

**Table 5:** Direct, indirect, and total effects of variables that influence perceived usefulness (PU) and people’s intention to use mHealth (INT).

Relationship	Direct	Indirect	Total
Health consciousness (HC) on PU	0.33	0.00	0.33
Communication effectiveness (CE) on PU	0.43	0.00	0.43
Perceived ease of use (PEOU) on PU	0.56	0.00	0.56
HC on INT	0.14	0.24	0.38
CE on INT	0.16	0.32	0.48
PEOU on INT	−0.13	0.42	0.29
PU on INT	0.74	0.00	0.74
DIST on INT	−0.07	0.00	−0.07

**Table 6:** The results of hypothesis testing.

Hypothesis	Path (Direction)	Standardized Coefficient ^a^	Supported
H1	PEOU → INT (+)	−0.13 *	No ^b^
H2	PU → INT (+)	0.74 ***	Yes
H3	PEOU → PU (+)	0.56 ***	Yes
H4	HC → PU (+)	0.33 ***	Yes
H5	HC → INT (+)	0.14 **	Yes
H6	CE → PU (+)	0.43 ***	Yes
H7	CE → INT (+)	0.16 **	Yes
H8	DIS → INT (-)	−0.07	No

^a^: * *p* < 0.05; ** *p* < 0.01; *** *p* < 0.001; ^b^: Although the direct path between PEOU and INT had a standardized coefficient of −0.13, the total effect of PEOU on INT was found to be 0.29 because PEOU had an indirect effect on INT with a magnitude of 0.42 through PU.
